# Mediators of allergic rhinitis: optimization of RNA isolation, reverse transcription, and qPCR protocols

**DOI:** 10.1186/1710-1492-10-S2-A64

**Published:** 2014-12-18

**Authors:** Caroline Conway, Jenny Thiele, Mena Soliman, Anne Ellis

**Affiliations:** 1 Department of Biomedical and Molecular Sciences, Queen’s University, Kingston, Ontario, K7L 3N6, Canada; 2 Department of Medicine, Queen’s University, Kingston, Ontario, K7L 3N6, Canada; 3 Division of Allergy & Immunology, Kingston General Hospital, Kingston, Ontario, K7L 2V7, Canada

## Background

Optimizing methods for the study of allergic rhinitis (AR), especially when using samples likely containing small amounts of material for analysis, ensures the integrity of results that may potentially enhance the understanding of AR disease mechanisms. In order to conduct future mRNA expression analysis, examining the differential expression of AR mediators such as *IL33*, *TSLPR*, *HPGDS*, and *CRTH2* at baseline and 6 hours following Nasal Allergen Challenge (NAC) in allergic individuals, this study aims to optimize the RNA isolation, reverse transcription (RT), and qPCR protocols used for the study of nasal mucosal samples.

## Methods

Several RNA isolation and RT kits were evaluated using nasal scrapings from healthy individuals, similar to those collected from allergic participants. These kits were evaluated based on the yield and purity of RNA and cDNA, assessed using spectrophotometry, qPCR amplification, and gel electrophoresis. Reference gene analysis using cDNA isolated from allergic participants was conducted using qPCR and the statistical software GenEx (MultID). Primer design and evaluation of primers for the targets of interest—*IL33*, *TSLPR*, *HPGDS*, and *CRTH2*—was also pursued.

## Results

RNA isolation and RT kit optimization determined that the Life Technologies-Qiagen (LT-Q) kit combination produced cDNA with maximal purity and qPCR efficiency compared with the other kit combinations evaluated. Reference gene analysis demonstrated that expression of ubiquitin C (UBC) showed limited variability among the differing conditions (time point and study) of nasal sample collection. Primer evaluation yielded inconsistent results.

## Conclusions

Future processing of nasal scraping samples should use the optimal LT-Q kit combination. Following successful primer evaluation, the expression levels of the targets of interest in the allergic nasal mucosal cDNA samples at both baseline and 6h post-NAC will be conducted via the optimized qPCR reaction, using UBC as a reference gene.

